# Unlocking Fertility: How Nitric Oxide Pathways Connect Obesity and Reproductive Health—The Role of Bariatric Surgery

**DOI:** 10.3390/antiox14020240

**Published:** 2025-02-19

**Authors:** Charalampos Voros, Despoina Mavrogianni, Kyriakos Bananis, Antonia Varthaliti, Anthi-Maria Papahliou, Vasileios Topalis, Panagiota Kondili, Menelaos Darlas, Maria Anastasia Daskalaki, Agni Pantou, Diamantis Athanasiou, Dimitris Mathiopoulos, Marianna Theodora, Panagiotis Antsaklis, Dimitrios Loutradis, Georgios Daskalakis

**Affiliations:** 1 1st Department of Obstetrics and Gynecology, ‘Alexandra’ General Hospital, National and Kapodistrian University of Athens, 80 Vasilissis Sofias Avenue, 11528 Athens, Greece; depy.mavrogianni@yahoo.com (D.M.); antonia.varthaliti@hotmail.com (A.V.); anthipapahliou@gmail.com (A.-M.P.); giotakondyli27@gmail.com (P.K.); mdarlas2110@gmail.com (M.D.); agni.pantos@gmail.com (A.P.); martheodr@gmail.com (M.T.); panosant@gmail.com (P.A.); gdaskalakis@yahoo.com (G.D.); 2 King’s College Hospitals NHS Foundation Trust, London SE5 9RS, UK; kyriakos.bananis@nhs.net; 3 Department of Internal Medicine, Hospital of Thun, 3600 Thun, Switzerland; vtopalismd@gmail.com; 4 IVF Athens Reproduction Center V. Athanasiou, 151 23 Maroussi, Greece; diamathan16@gmail.com; 5 Rea Maternity Hospital S.A., Avenue Siggrou 383 &Pentelis 17, P. Faliro, 17564 Athens, Greece; dimitrismathiopoulos@gmail.com; 6 Fertility Institute-Assisted Reproduction Unit, Paster 15, 11528 Athens, Greece; loutradi@otenet.gr; 7 Athens Medical School, National and Kapodistrian University of Athens, 15772 Athens, Greece

**Keywords:** infertility, bariatric surgery, CART peptide, leptin, ENOS, oxidate stress

## Abstract

This study examines the relationship between obesity, oxidative stress, and reproductive dysfunction. It focuses on the effects of sleeve gastrectomy on gene expression and hormone profiles in 29 women with severe obesity (BMI ≥ 40 kg/m^2^). Pre- and post-surgical investigations revealed significant differences in major gene expressions and hormonal markers. CART expression reduced significantly from 0.27 ± 4.43 to −3.42 ± 1.14 (*p* < 0.001), while leptin expression decreased from −1.87 ± 1.75 to −0.13 ± 1.55 (*p* < 0.001), indicating better metabolic regulation. In contrast, eNOS expression increased considerably from −4.87 ± 1.70 to 1.18 ± 2.31 (*p* = 0.003), indicating improved endothelial function and nitric oxide bioavailability, which is critical for vascular health and reproduction. Correlation research before surgery indicated no significant relationships between eNOS, CART, or leptin and clinical indicators, implying that these genes function independently in pre-surgical metabolism. While most associations remained negligible after surgery, a significant negative connection between eNOS expression and SHBG levels appeared (r = −0.365, *p* = 0.049), indicating potential interactions in hormonal regulation pathways following metabolic improvements. These findings emphasize the importance of bariatric surgery in reducing the negative effects of obesity on reproductive health by altering critical cellular pathways. Significant increases in CART, leptin, and eNOS expression indicate reduced oxidative stress, improved vascular tone, and hormonal balance, all of which contribute to increased reproductive capacity. This study sheds light on the molecular processes that link obesity, metabolic health, and fertility, underlining bariatric surgery’s therapeutic potential for women experiencing obesity-related infertility.

## 1. Introduction

Overweight and obesity have become more common in recent years as a result of dietary and lifestyle changes. In the United States, more than 30% of the population is currently considered obese [1,2]. The frequency varies worldwide, but in industrialized countries, a sizable proportion of the population is afflicted. In America, around one-third of people have a BMI exceeding 30 kg/m^2^ [3]. Obesity is considered the second most important cause of death after smoking. According to Roland Sturm, the healthcare costs associated with obesity outweigh those related to smoking [4]. Obesity is observed not only in adults but also in younger ages because of excessive sugary beverage intake and prolonged screen time [5,6].

Obesity is a significant public health concern in Greece, with current data showing that 38% of adults are overweight and 32% are obese. Alarmingly, childhood obesity rates are among the highest in Europe, with 18% of children aged 7 to 10 being obese. These changes emphasize the critical need for focused interventions to combat obesity and its associated health risks [7].

Obesity is a complicated metabolic condition that has a substantial influence on reproductive health, especially in women. The disorder is linked to endocrine disruption, chronic inflammation, and vascular dysfunction, which impairs ovulation and endometrial receptivity [8].

Although elevated leptin levels in obesity are supposed to lower food intake and increase energy expenditure, “leptin resistance” confuses this relationship. This resistance results from reduced leptin transport across the blood–brain barrier and altered intracellular signaling pathways, which are frequently exacerbated by chronic inflammation associated with obesity, emphasizing leptin’s complicated function in reproductive physiology [9]. In obesity, leptin dysregulation impairs not only gonadotropin-releasing hormone (GnRH) secretion but also the production of fertility-related hormones, potentially leading to infertility [10]. Furthermore, the leptin receptors found in ovarian tissue directly influence follicular growth [11]. At the molecular level, leptin has been found to activate the JAK-STAT and PI3K-Akt signaling pathways, which affect downstream targets such as the cocaine- and amphetamine-regulated transcript (CART) neuropeptide in the central nervous system. This connection points to a considerable interplay between leptin and CART in the control of energy balance and reproductive function [11]. Obese individuals’ granulosa cells (GCs) have also been found to have elevated CART levels. Leptin signaling induces CART expression, which inhibits important enzymes such as aromatase via transcriptional repression mediated by intracellular signaling cascades such as the CREB and MAPK pathways. This inhibition decreases steroidogenesis, which further impairs ovarian function [12]. These molecular interactions highlight the complex role of leptin–CART crosstalk in linking metabolic disorders and reproductive health.

Endothelial nitric oxide synthase (eNOS) has a major role in obesity-induced reproductive difficulties because it produces nitric oxide (NO), which is a fundamental regulator of vascular tone and cellular signaling. NO is a cellular messenger and effector molecule known for its simple structure, fast diffusion, and short biological half-life. As an inorganic free radical, NO acts as a unique biological signal molecule, impacting a variety of physiological and pathological processes [13,14].

In the reproductive system, NO is necessary for follicular development, oocyte maturation, ovulation, and embryo implantation [15]. In men, it has an effect on spermatogenesis and sperm capacitation [16]. However, with obesity, increasing oxidative stress depletes the necessary cofactor tetrahydrobiopterin (BH4), causing eNOS “uncoupling”, resulting in the formation of superoxide (O^2−^) rather than NO [17]. This shift produces damaging oxidative byproducts such as peroxynitrite (ONOO-), which causes cellular damage and reduces NO bioavailability [18].

Elevated NO_2_ (nitrite) and NO_3_ (nitrate) levels indicate abnormal NO metabolism and decreased vascular function, affecting blood flow and cellular communication in the ovaries and uterus [19]. These molecular alterations have a negative impact on folliculogenesis, oocyte quality, and embryo implantation, and are associated with disorders such as polycystic ovarian syndrome (PCOS), which contributes to infertility [20]. Bariatric surgery increases NO bioavailability by lowering oxidative stress and restoring eNOS activity, with benefits for vascular health and reproductive outcomes.

The present study investigates the relationship between obesity, oxidative stress, and infertility through the expression of CART, leptin, and eNOS genes, focusing on the therapeutic potential of bariatric surgery in restoring reproductive health.

## 2. Materials and Methods

The study included 29 women under 40 with a BMI of 40 kg/m^2^ or above, according to the Helsinki Declaration ethical norms. The Alexandra General Hospital (Approval No. 4345, Date: 1 April 2023) provided the ethical approval. All participants gave their informed consent for this prospective study, which was realized from March 2022 to November 2024 at the First Department of Obstetrics and Gynecology of ‘Alexandra’ General Hospital. Infertility was defined as the failure to produce clinical pregnancy following a year of regular, unprotected sexual intercourse.

**Figure 1:**
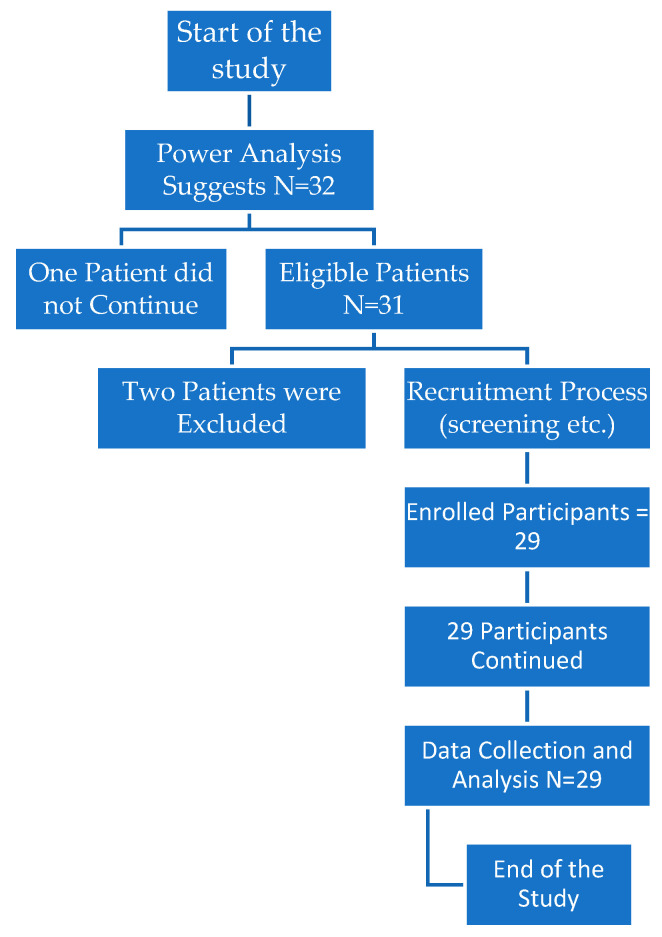
Flowchart of patients’ elimination and inclusion in the study.

Participants were chosen based on their BMI and infertility diagnosis, and to ensure homogeneity, those with serious medical disorders, those on fertility-affecting drugs, and those over 40 were excluded. Consequently, confounding factors which might affect gene expression or reproductive outcome were excluded. Following a sleeve gastrectomy, patients were generally hospitalized for 1–2 days before beginning a gradual nutritional transition from liquids to solids. The rigorous inclusion criteria, ethical concerns, and comprehensive follow-up ensure that the study findings are legitimate. Figure 1 shows the study’s design and participant flow.

The flowchart in Figure 1 depicts the study’s systematic approach to participant recruiting, screening, and data collecting. Initially, a power analysis yielded a desired sample size of 32 individuals. During recruiting, 31 patients were recognized as eligible, however one chose not to proceed. As a result, 29 individuals were included in the trial, with two patients rejected due to particular criteria. The recruiting step includes a rigorous screening to verify that all participants satisfied the study’s eligibility criteria. Although the initial power analysis suggested 32 participants, the final sample size of 29 was considered enough to proceed with data collection and analysis. Following recruiting, the study collected and evaluated data from the remaining 29 individuals, leading to the study’s conclusion. The flowchart successfully demonstrates how the study handled participant exclusions and ensured adherence to criteria, while still maintaining a sample size close to the recommended target.

### 2.1. Hormone Measurements

Blood samples have been obtained from participants before and six months after sleeve gastrectomy. Serum hormone levels were determined using chemiluminescent immunoassays (CLIAs) on the Roche Cobas e411 analyzer (Roche Diagnostics, Basel, Switzerland). Standardization was based on the following hormone reference ranges: the follicle-stimulating hormone (FSH) 3.5–12.5 mIU/mL, luteinizing hormone (LH) 2.4–12.6 mIU/mL, estradiol (E2) 19.5–144.2 pg/mL, sex hormone-binding globulin (SHBG) 18–144 nmol/L, anti-Müllerian hormone (AMH) 1.0–8.0 ng/mL, and free testosterone 0.3–3.5 ng/dL. These reference values were established using the manufacturer’s guidelines and the relevant clinical literature.

### 2.2. Detection of Leptin, Oxidative Stress Markers, and Gene Expression

Total RNA was extracted from blood samples using New England Biolabs Monarch Total RNA Miniprep Kit (Ipswich, MA, USA). After extracting RNA, 1 μg was utilized to synthesize complementary DNA (cDNA) using New England Biolabs Luna Script RT SuperMix. To measure gene expression, 5 μL of produced cDNA was used in RT-PCR experiments.

All RT-PCR reactions were conducted using the Light Cycler 480 Instrument II from Roche Life Sciences, using the Luna Universal qPCR Master Mix (New England Biolabs) at a final concentration of 1×. The Luna Universal qPCR Master Mix (part number M3003L, New England Biolabs, Ipswich, MA, USA) was used for real-time quantitative PCR (qPCR). Eurofins Genomics generated the primers for CART, leptin, endothelial nitric oxide synthase (eNOS), and glucose-6-phosphate dehydrogenase (G6PD) (housekeeping gene). The following primer sequences were used: CART (Forward: 5′-GCTGCTGGACATCCTGCTAA-3′, Reverse: 5′-GCTGCTGGACATCCTGCTAA-3′), leptin (Forward: 5′-ACCCTTCCAAACCCTCAG-3′, Reverse: 5′-GAGGAGGTCTCGCAGGTTCT-3′), eNOS (Forward: 5′-CAGTGTCCAACATGCTGCTGGAA-3′, Reverse: 5′-GCCCGGCTGTCATGGTGCTG-3′), and G6PD. These primer sequences were chosen based on previously confirmed qPCR tests and optimized for specificity and efficiency in human samples. ΔΔCT computations used pre-surgical (baseline) samples from each patient as the internal reference, allowing for the intra-individual normalization of post-surgery gene expression changes. This normalizing strategy reduced inter-individual variability, resulting in the precise estimation of relative gene expression changes before and after bariatric surgery. The RT-PCR technique consisted of an initial denaturation phase at 94 °C for one minute, followed by 40 cycles of denaturation at 95 °C for 15 s and annealing/extension at 60 °C for 30 s. To confirm data specificity, a melting curve study was conducted. To standardize gene expression levels, the internal housekeeping gene G6PD was used. Each experiment was duplicated to verify data accuracy and dependability. In addition, a negative control was used to account for any potential contamination or background signal. The relative mRNA expression levels of all genes were determined using the 2^−ΔΔCT^ technique.

To normalize gene expression variations after surgery, ΔΔCT calculations employed pre-surgical (baseline) samples from each patient as an internal control. This technique guarantees that each patient’s gene expression levels are compared to their own pre-surgical baseline, reducing inter-individual variability.

Gene expression analysis was carried out on whole blood samples. Total RNA was isolated from whole blood using the New England Biolabs Monarch Total RNA Miniprep Kit (Ipswich, MA, USA). Whole blood was chosen for its ability to represent systemic gene expression changes, particularly for genes implicated in metabolic and hormonal regulation, such as eNOS, CART, and leptin. To provide clarity, the gene expression results are labeled as follows: Cpt0 represents baseline gene expression levels (before surgery), whereas Cpt1 represents post-surgical gene expression levels (six months later). For example, Cpt0 leptin represents leptin expression at baseline, whereas Cpt1 CART reflects CART expression after surgery.

### 2.3. Statistical Analysis

Data were systematically recorded in Microsoft Excel (version 2401, 2016) spreadsheets (Microsoft Corporation, Redmond, Washington, DC, USA), with each row representing a single patient. Statistical analyses were performed with the SAS software platform for Windows (version 9.4, SAS Institute Inc., Cary, NC, USA). Descriptive data were reported as mean values with standard deviations (SDs). To examine hormone level changes, the difference was computed as the post-surgical value minus the pre-surgical value, with a positive value indicating a rise and a negative value indicating a drop. The nonparametric Wilcoxon signed-rank test was used to compare the matched data. Additionally, the number and percentage of patients who had a rise or reduction in hormone levels were calculated. To investigate the association between changes in hormone levels, changes in body mass index (BMI), and gene expression levels, the Spearman correlation coefficient (rs) was obtained. Correlations between variables were determined using the Spearman correlation test, as the Shapiro–Wilk test revealed deviations from normality in the data. A positive rs value near 1 implies a high positive connection, whereas a negative rs value closer to −1 indicates a strong negative link. The significance level was set at *p* < 0.05, with all statistical tests being two-sided. The paired *t*-test was employed to compare pre-surgical and post-surgical gene expression levels (CART, leptin, and eNOS), since the Shapiro–Wilk test verified the changes to be approximately normal. These comparisons are given using the t-statistic and the related *p*-values.

### 2.4. Sample Size Determination

A power analysis was performed to determine the optimum sample size for this investigation, guaranteeing adequate statistical power to detect significant differences in the important outcomes. To calculate the effect size for our research, we thoroughly evaluated the current literature and pertinent studies, as well as sought expert input. The two key outcome variables, ENOS, CART, and leptin gene expression, were anticipated to have a modest impact size. This impact size was regarded as a conservative estimate that would allow for the detection of significant changes in gene expression.

We used a statistical power objective of 80% (1 − β = 0.80) to increase the likelihood of detecting actual effects. The significance threshold (α) was set at 0.05, representing a 5% chance of making a type I mistake. Paired *t*-tests were performed to assess the average expression levels of ENOS, CART, and leptin genes before and six months after bariatric surgery. For nonparametric analysis of paired data, the Wilcoxon signed-rank test was used. In addition, based on comparable research, we assumed a modest correlation value of 0.5 to account for the link between pre- and post-surgery parameters.

The statistical program G*Power (version 3.1.9.7) was used to perform the power analysis with these parameters. The results showed that a total sample size of 29 individuals was necessary to obtain the appropriate statistical power. To account for probable attrition, an estimated sample size of 32 participants was advised, assuming a 10% loss to follow-up. Practical concerns such as patient availability and budget restrictions were also assessed to ensure the study’s viability in a clinical environment.

## 3. Results

Table 1 shows that a correlation analysis between eNOS and several clinical indicators was examined prior to bariatric surgery. The mean age was 33.03 years (SD = 4.08), with a Pearson correlation coefficient of 0.111 (*p* = 0.565). For hormonal parameters, FSH had a mean of 6.03 mIU/mL (SD = 1.09) and a correlation of 0.188 (*p* = 0.328), whereas LH had a mean of 6.87 mIU/mL (SD = 1.01) and an insignificant correlation of 0 (*p* = 0.997). Estradiol (E2) levels averaged 30.75 pg/mL (SD = 3.80), with a minor correlation of 0.249 (*p* = 0.193), whereas SHBG levels averaged 36.24 nmol/L (SD = 7.58), with a slight negative correlation of −0.076 (*p* = 0.696). Free testosterone exhibited the greatest variability (mean = 28.72 ng/dL, SD = 9.35) and a slight positive correlation of 0.285 (*p* = 0.133). AMH levels averaged 2.14 ng/mL (SD = 0.24), with a correlation coefficient of 0.115 (*p* = 0.552). For ovarian reserve markers, AFC in the right ovary (mean = 10.31, SD = 2.51) and left ovary (mean = 9.28, SD = 2.59) had associations of 0.095 (*p* = 0.623) and 0.063 (*p* = 0.746), respectively. Finally, the mean BMI before surgery was 41.12 (SD = 3.28), with a negative correlation of −0.266 (*p* = 0.162). Overall, none of these relationships was statistically significant (all *p* > 0.05), indicating that eNOS gene expression is not linearly linked with these clinical indicators prior to bariatric surgery.

Table 2 indicates the association of eNOS gene expression and several clinical indicators following bariatric surgery. The mean AFC in the left ovary after surgery was 6.76 (SD = 1.24), with a Pearson correlation of −0.149 (*p* = 0.439), and in the right ovary, 7.38 (SD = 1.64), with a correlation of 0.037 (*p* = 0.849). AMH levels after surgery averaged 2.96 ng/mL (SD = 0.15), with a correlation coefficient of 0.102 (*p* = 0.597). Six months after surgery, the BMI averaged 25.98 (SD = 1.38), with a negative correlation of −0.248 (*p* = 0.194). Estradiol (E2) levels averaged 49.23 pg/mL (SD = 4.54), with a correlation of −0.156 (*p* = 0.42), whereas FSH levels post-surgery averaged 8.88 mIU/mL (SD = 0.08), with a weak correlation of 0.08 (*p* = 0.677). Free testosterone levels averaged 9.42 ng/dL (SD = 2.52) and showed a weak negative correlation of −0.151 (*p* = 0.436). LH levels after surgery averaged 8.83 mIU/mL (SD = 1.03), with a −0.121 correlation (*p* = 0.532). SHBG had the strongest connection (−0.365, *p* = 0.049), demonstrating a small but statistically significant negative link with Cpt eNOS. Overall, only SHBG shows a possible association with gene expression post surgery.

Table 3 shows the descriptive statistics and the correlation analysis of gene expression parameters prior to surgery. The mean expression of eNOS was −4.85 (SD = 1.75), with values ranging from −7.52 to −0.23, whereas CART had a mean of 0.27 (SD = 4.43) and ranged from −4.1 to 10.72, indicating greater variability. Leptin had a mean of −1.87 (SD = 1.75), with a range of −5.5 to 1.09.

Regarding Table 4, the connection between eNOS and CART (r = 0.038, *p* = 0.848) indicates no significant link. Similarly, the connection between Cpt eNOS A and Cpt0 leptin was negative and modest (r = −0.301), but not statistically significant (*p* = 0.12). The correlation between Cpt0 CART and Cpt0 leptin was equally minimal (r = 0.077, *p* = 0.697). Overall, none of the relationships were statistically significant (all *p*-values more than 0.05), indicating that these gene expressions are not linearly related prior to surgery.

Following surgery in Table 5, descriptive statistics and correlation analysis of gene expression characteristics reveal modest relationships between Cpt1eNOS, Cpt1 CART, and Cpt1 leptin. The average expression of Cpt1eNOS was 1.18 (SD = 2.31), with values ranging from −2.07 to 6.77. Cpt1 CART had a mean of −3.4 (SD = 1.12) and ranged from −5.78 to −0.74, whereas Cpt1 leptin had a mean of −0.07 (SD = 1.55) and ranged from −3.34 to 2.64.

Table 6 indicates that the relationship between eNOS and CART was minimal (r = 0.16) and not statistically significant (*p* = 0.40), indicating no relevant relationship. Similarly, the correlation between Cpt1eNOS and Cpt1 leptin was modest (r = 0.039) and had a high *p*-value (*p* = 0.838), indicating no meaningful relationship. Cpt1 CART and Cpt1 leptin showed a weak positive connection (r = 0.282), however it was not statistically significant (*p* = 0.136). Overall, none of the correlations were statistically significant (all *p*-values > 0.05), indicating that there are no substantial linear connections between these gene expressions following surgery.

Table 7 summarizes the changes in gene expression for CART, leptin, and eNOS before and after bariatric surgery, including mean values, standard deviations, correlations, and statistical significance. The mean expression of CART fell considerably from 0.273 (SD = 4.43) before surgery to −3.42 (SD = 1.14) after surgery, with a t-statistic of 4.52 and a highly significant *p*-value of <0.001. Leptin expression decreased significantly from −1.87 (SD = 1.75) before surgery to −0.130 (SD = 1.55) after surgery, with a t-statistic of −7.35 and a *p*-value of <0.001. Following surgery, eNOS expression increased from a mean of −4.87 (SD = 1.7) to 1.18 (SD = 2.31), with a moderate positive correlation (r = 0.529) and a statistically significant *p*-value (*p* = 0.003), indicating a substantial relationship in expression changes before and after surgery.

**Scheme 1:**
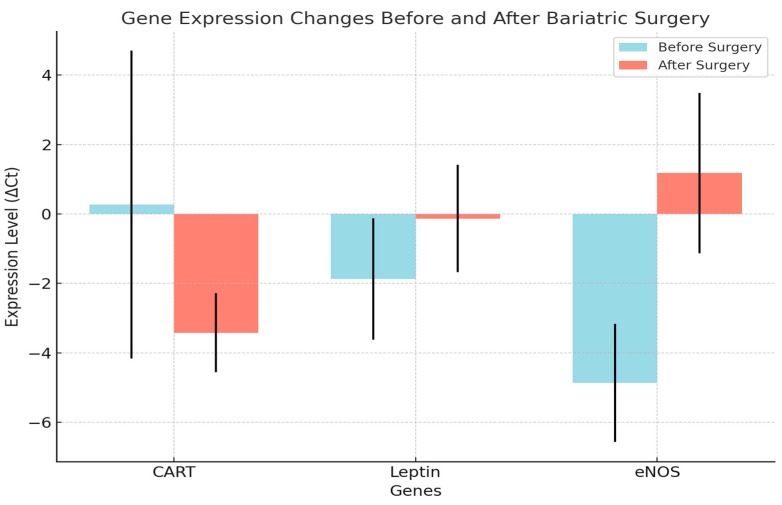
Gene expression changes before and after bariatric surgery.

Scheme 1 shows the comparison of gene expression levels (CART, leptin, and eNOS) before and after bariatric surgery. The graphic shows the mean expression levels (ΔCt), with error bars showing standard deviations. The blue markers (circles) represent pre-surgical expression levels, whereas the red markers (squares) represent post-operative expression levels. Following surgery, CART and leptin expression levels are reduced, but eNOS levels increase significantly. These alterations indicate metabolic and hormonal adjustments after weight reduction. Statistical significance is shown in the findings section.

## 4. Discussion

This study glanced at the link between BMI alterations after bariatric surgery, hormone profiles, and gene expression in infertile women. A significant increase in eNOS expression after surgery (*p* = 0.003) indicates enhanced endothelial function and vascular health, most likely due to lower oxidative stress and inflammation associated with weight loss. Furthermore, sex hormone-binding globulin (SHBG) had a strong negative connection with eNOS expression (r = −0.365, *p* = 0.049), implying a potential involvement for SHBG in hormonal control pathways following metabolic improvements.

Bariatric surgery significantly reduced CART and leptin expression (*p* < 0.001), indicating metabolic changes to weight loss, including improved energy balance and leptin sensitivity. These modifications underscore CART and leptin’s roles in hunger and energy regulation. While no strong interdependence was found amongst the examined genes, these data indicate that eNOS, CART, and leptin may be useful biomarkers for metabolic and reproductive recovery after surgery.

Obesity remedies include both nonsurgical and surgical therapies, with bariatric surgery being particularly beneficial in improving metabolic health, reducing cardiovascular risks, and dealing with obesity-induced infertility [21,22,23]. The surgery used in this study, sleeve gastrectomy, decreases the stomach size to a tubular shape, restricting food intake and causing major hormonal and metabolic alterations. These include decreases in ghrelin and increases in satiety hormones, contributing to significant weight loss and improvements in energy balance [24,25].

Nitric oxide is involved in several reproductive processes in female animals, including follicular development, ovulation, oocyte maturation, and luteal function [15]. The enzyme nitric oxide synthase produces NO, and its expression in ovarian follicles varies by species and stage of development [26].

Adipose tissue is no longer thought of as a passive storage location for free fatty acids (FFAs), but rather as a dynamic endocrine organ that secretes a variety of mediators known as adipokines. These proteins include TNF-α, resistin, IL-6, ASP, AGT, PAI-1, leptin, and adiponectin. They are involved in several metabolic processes, including energy balance, food intake, fat metabolism, vascular tone modulation, and insulin sensitivity [27,28]. Although leptin and adiponectin help regulate energy and metabolism, other variables such as TNF-α, IL-6, and resistin can worsen insulin resistance. Angiotensinogen and PAI-1 are linked to vascular problems associated with obesity [29].

Leptin, a 16-kDa hormone largely generated in adipose tissue, is critical in hunger control. Obesity has been associated to mutations in the leptin and leptin receptor genes [29,30]. In obesity, oxidative stress can be caused by a variety of factors, including hyperglycemia, hyperleptinemia, insufficient antioxidant defenses, increased lipid levels in muscle, increased muscle activity, elevated free radical production rates, mitochondrial dysfunction, endothelial impairment, and chronic inflammation [31,32].

CART expression is important in morbid obesity and is closely linked to hormonal pathways, as evidenced by animal research. Asnicar et al. studied CART-deficient mice and discovered that they were more prone to obesity when fed a high-fat diet [24]. Similarly, Lee et al. found that injecting CART into the nucleus tractus solitarii reduced food consumption in obese rats [33]. Kristensen et al. demonstrated that CART peptides inhibited feeding behavior even in the presence of neuropeptide Y (NPY), a powerful appetite stimulant in the central nervous system [11]. Furthermore, Okumura et al. and Asakawa et al. found that CART injection into the fourth ventricle delayed stomach emptying, similar to the effects of the corticotropin-releasing factor (CRF) and cholecystokinin [34,35]. Patkar et al. investigated the link between bariatric surgery and CART expression in a mouse model of morbid obesity. Mice were separated into the following four groups based on the intervention: Roux-en-Y gastric bypass (RYGB), sleeve gastrectomy, calorie restriction, and a control group receiving no intervention. The study found no statistically significant differences in CART mRNA expression among the groups. Furthermore, the findings indicated that RYGB had distinct effects on hypothalamic gene regulation in comparison to calorie restriction or weight loss alone [36]. Grayson et al. and Cavin et al. found no significant changes in CART expression following bariatric surgery in obese rats, which supports these findings [37,38]. Patkar et al. confirmed these findings, revealing no quantifiable changes in CART levels before and after RYGB, nor any variations in CART mRNA expression between chow-fed and RYGB-treated mice [36]. Muñoz-Rodríguez et al. found that obese patients had higher CART levels before bariatric surgery than those with normal weight, but there was no significant difference one year later [39]. In their 2021 assessment, Singh et al. suggested that the CART peptide has potential as a treatment agent for morbid obesity [40].

In cattle, eNOS is present in granulosa cells at all stages of follicular development [41]. In porcine, eNOS is expressed in big follicles (7–10 mm), but not in medium-sized follicles (3–6 mm) [42]. These findings highlight the species-specific expression patterns of NOS and point to a distinct functional role of NO in ovarian physiology.

NO also regulates the growth and development of preantral follicles. NO levels vary throughout follicular development, indicating that it influences follicular expansion and atresia [43]. In preantral follicle investigations, enhanced eNOS expression was detected in response to gonadotropins such as human chorionic gonadotropin (hCG), emphasizing its role in follicular development and differentiation [44,45]. The dual role of NO in follicular development may be due to its vasodilatory impact, which influences follicular blood flow and nutrient delivery [46].

Experiments involving mice treated with NOS inhibitors or eNOS mutant animals demonstrate that NO plays a function in oocyte maturation [47]. These oocyte meiosis defects indicate that the cumulus–oocyte complex development is dependent on the NO/NOS system [48]. Furthermore, NO has a concentration-dependent effect, with low levels encouraging oocyte maturation and high levels inhibiting it via decreasing estradiol (E2) production [49]. This underscores the need of precisely regulating NO levels in order to ensure optimum oocyte maturation.

During ovulation, NO is involved in vascular remodeling and follicular rupture. Non-specific NOS inhibitors, such as aminoguanidine (AG) and L-NAME, have been demonstrated in studies to drastically lower ovulation rates, which may be reversed by NO donors such as sodium nitroprusside (SNP) [50]. The control of NO during ovulation involves complicated hormonal interactions, such as increasing luteinizing hormone (LH) production to stimulate preovulatory processes [51]. Furthermore, NO has been demonstrated to influence the synthesis of E2, which governs ovulation and follicular development.

NO also regulates follicular atresia by inhibiting granulosa cell death. It has been shown that iNOS-derived NO inhibits apoptosis in rat granulosa cells via autocrine and paracrine signaling, preventing early follicular atresia [41,52]. High NO levels, on the other hand, can induce apoptosis in well-differentiated granulosa cells, demonstrating a dual regulatory role dependent on concentration and follicular stage [53]. NO regulates apoptosis through mechanisms such as the Fas/FasL system and the modulation of pro- and anti-apoptotic gene expression [54]. In fertilization, NO regulates oocyte activation by affecting intracellular calcium Ca^2^⁺ (calcium ion), signaling, and sperm capacitation [55]. NO’s role in these activities is critical for proper fertilization and early embryo development. Furthermore, NO regulates embryo implantation and pregnancy maintenance by creating a favorable uterine environment, as well as modulating uterine contractility and placental blood flow [56,57]. NO also helps to maintain vascular resistance and promote nutrient exchange, both of which are necessary for fetal growth and development [58].

Although an association between eNOS, CART, and leptin is not established, they should be considered as putative biomarkers for fertility outcome in obese women. Additionally, it could be of great interest to further examine a possible parallel action of miRNAs, regulating the expression of the aforementioned genes. Both the regulation of oxidative stress and metabolic disorders through the creation of a related to them genetic profile may be of great value to fertility reestablishment after bariatric surgery.

## 5. Study Limitations

This study has various limitations that should be acknowledged. First, our findings may be limited in their generalizability due to the relatively small sample size (*n* = 29). While the data show significant changes in gene expression and hormone profiles after bariatric surgery, larger research is needed to confirm these findings. Second, while bariatric surgery was the primary intervention, the observed improvements in gene expression and hormonal balance could have been impacted by lifestyle modifications, dietary changes, and increased physical activity, all of which are frequent components of post-surgical care. These characteristics were not independently examined in this investigation and may have influenced the overall results. In addition, because the study focuses exclusively on surgical treatment, it does not thoroughly investigate nonsurgical treatments such as rigorous lifestyle modifications or pharmaceutical therapies, which may possibly play a role in treating obesity-related infertility. Future research comparing the efficacy of different modalities would provide a more comprehensive picture of the available therapy options.

## 6. Conclusions

Bariatric surgery causes major metabolic and hormonal changes, including changes in nitric oxide (NO) bioavailability, leptin signaling, and CART expression, all of which play important roles in energy balance, vascular function, and reproductive physiology. The observed increase in eNOS expression after surgery implies a possible improvement in endothelial function, which might promote ovarian blood flow and follicular growth. Furthermore, the downregulation of leptin and CART expression is consistent with prior research indicating that high leptin and CART activity may impair gonadotropin production and ovarian function in obese persons. These molecular and hormonal changes emphasize the potential physiological benefits of weight loss in those suffering from obesity-related infertility.

However, while our data show significant changes in hormone profiles and gene expression, they do not indicate a clear causal association between bariatric surgery and better reproductive outcomes. The lack of direct clinical fertility evaluations, such as ovulation monitoring, implantation rates, and pregnancy outcomes, restricts our capacity to evaluate whether the observed metabolic and hormonal changes lead to increased fertility. Furthermore, it is unknown whether the effects of bariatric surgery on NO bioavailability and hormonal balance are long-lasting or if other variables, such as dietary status and lifestyle changes, contribute to these changes.

Future research should focus on longitudinal fertility assessments that include clinical reproductive markers such as menstrual cycle regularity, antral follicle count (AFC) post-surgery, endometrial receptivity markers, and pregnancy outcomes in women undergoing assisted reproductive technologies (ARTs). Furthermore, exploring possible epigenetic and microRNA-mediated processes that regulate the expression of CART, leptin, and eNOS may reveal further information on the molecular interactions between obesity, metabolic regulation, and reproductive health.

While bariatric surgery tends to provide beneficial metabolic and hormonal changes, its direct influence on reproductive outcomes is not entirely understood. Our findings suggest a potential role for NO bioavailability in reproductive adaptation following weight reduction. However, more studies are needed to identify whether these molecular alterations directly translate into better fertility and pregnancy success rates in those women.

## Figures and Tables

**Table 1:** Correlation analysis of eNOS expression with clinical parameters before bariatric surgery.

Variable	Mean ± SD	Pearson Correlation (r)	*p*-Value
Age (years)	33.03 ± 4.08	0.111	0.565
FSH (mIU/mL)	6.03 ± 1.09	0.188	0.328
LH (mIU/mL)	6.87 ± 1.01	0.000	0.997
Estradiol (E2, pg/mL)	30.75 ± 3.80	0.249	0.193
Sex Hormone-Binding Globulin (SHBG, nmol/L)	36.24 ± 7.58	−0.076	0.696
Free Testosterone (ng/dL)	28.72 ± 9.35	0.285	0.133
Anti-Müllerian Hormone (AMH, ng/mL)	2.14 ± 0.24	0.115	0.552
Antral Follicle Count—Right Ovary (AFC)	10.31 ± 2.51	0.095	0.623
Antral Follicle Count—Left Ovary (AFC)	9.28 ± 2.59	0.063	0.746
Body Mass Index (BMI, kg/m^2^) (Before Surgery)	41.12 ± 3.28	−0.266	0.162

The table shows the mean values, standard deviations, Pearson correlation coefficients, and *p*-values for each parameter. Follicle-Stimulating Hormone (FSH), Luteinizing Hormone (LH), Estradiol (E2), Sex Hormone-Binding Globulin (SHBG), Anti-Müllerian Hormone (AMH), Antral Follicle Count (AFC), and Body Mass Index (BMI). The Pearson correlation (r) values show the degree and direction of the link between eNOS expression and each parameter.

**Table 2:** Correlation analysis of Cpt eNOS and clinical parameters following bariatric surgery.

Variable	Mean ± SD	Pearson Correlation (r)	*p*-Value
Antral Follicle Count—Left Ovary (AFC)	6.76 ± 1.24	−0.149	0.439
Antral Follicle Count—Right Ovary (AFC)	7.38 ± 1.64	0.037	0.849
Anti-Müllerian Hormone (AMH, ng/mL)	2.96 ± 0.15	0.102	0.597
Body Mass Index (BMI, kg/m^2^) (After 6 Months)	25.90 ± 1.38	−0.248	0.194
Estradiol (E2, pg/mL)	49.2 ± 4.54	−0.156	0.420
Follicle-Stimulating Hormone (FSH, mIU/mL)	8.87 ± 0.08	0.080	0.677
Free Testosterone (ng/dL)	9.42 ± 2.52	−0.151	0.436
Luteinizing Hormone (LH, mIU/mL)	8.83 ± 1.03	−0.121	0.532
Sex Hormone-Binding Globulin (SHBG, nmol/L)	64.19 ± 12.41	**−0.365**	**0.049**

Abbreviations: AFC (Antral Follicle Count), AMH (Anti-Müllerian Hormone), BMI (Body Mass Index), E2 (Estradiol), FSH (Follicle-Stimulating Hormone), LH (Luteinizing Hormone), and SHBG (Sex Hormone-Binding Globulin). The Pearson correlation (r) values show the degree and direction of the link between eNOS expression and each parameter. The bolded values represent statistical significance (*p* < 0.05).

**Table 3:** Descriptive statistics and correlation analysis of gene expression parameters prior to surgery.

Statistic	eNOS	CART	Leptin
Count (n)	29	29	29
Mean ± SD	−4.85 ± 1.75	0.27 ± 4.43	−1.87 ± 1.75
Minimum	−7.52	−4.10	−5.50
25th Percentile	−6.19	−2.20	−2.72
Median	−5.01	−1.41	−2.02
75th Percentile	−3.38	0.08	−0.67
Maximum	−0.23	10.72	1.09

Abbreviations: eNOS (Endothelial Nitric Oxide Synthase); CART (Cocaine- and Amphetamine-Regulated Transcript). Whole blood samples were evaluated for gene expression levels prior to bariatric surgery. Values are shown as mean ± SD, with percentile distributions (minimum, 25th percentile, median, 75th percentile, and maximum).

**Table 4:** Correlation analysis of eNOS, CART, and leptin gene expression.

Gene	Correlation with Cpt0 CART	*p*-Value with Cpt0 CART	Correlation with Cpt0 Leptin	*p*-Value with Cpt0 Leptin
eNOS	0.038	0.848	−0.301	0.120
CART	-	-	0.077	0.697
Leptin	-	-	-	-

**Table 5:** Descriptive statistics and correlation analysis of gene expression parameters following surgery.

Statistic	Cpt1 eNOS	Cpt1 CART	Cpt1 Leptin
Count (n)	29	29	29
Mean ± SD	1.18 ± 2.31	−3.40 ± 1.12	−0.07 ± 1.55
Minimum	−2.07	−5.78	−3.34
25th Percentile	−0.78	−4.10	−1.08
Median	0.94	−3.70	0.00
75th Percentile	2.38	−2.77	1.05
Maximum	6.77	−0.74	2.64

Abbreviations: Cpt1 eNOS, Endothelial Nitric Oxide Synthase (post-surgery expression level); Cpt1 CART, Cocaine- and Amphetamine-Regulated Transcript (post-surgery expression level); Cpt1 leptin, leptin gene expression after surgery. Gene expression levels were measured in whole blood samples six months following bariatric surgery. Data are provided as mean ± SD and percentile distributions (minimum, 25th percentile, median, 75th percentile, and maximum).

**Table 6:** Correlation analysis of gene expression parameters following surgery.

Gene	Correlation with Cpt1 CART	*p*-Value with Cpt1 CART	Correlation with Cpt1 Leptin	*p*-Value with Cpt1 Leptin
Cpt1 eNOS	0.16	0.400	0.039	0.838
Cpt1 CART	—	—	0.282	0.136
Cpt1 Leptin	—	—	—	—

Abbreviations: Cpt1 eNOS, Endothelial Nitric Oxide Synthase (post-surgery expression level); Cpt1 CART, Cocaine- and Amphetamine-Regulated Transcript (post-surgery expression level); Cpt1 leptin, leptin gene expression after surgery. Pearson correlation coefficients (r) measure the degree and direction of the link between gene expression levels, while *p*-values show statistical significance.

**Table 7:** The mean gene expression values and standard deviations for CART, leptin, and eNOS before and after bariatric surgery.

Gene	Before Surgery (Mean ± SD)	After Surgery (Mean ± SD)	Correlation (r)	*p*-Value
CART	0.27 ± 4.43	−3.42 ± 1.14	4.52	<0.001
Leptin	−1.87 ± 1.75	−0.13 ± 1.55	−7.35	<0.001
eNOS	−4.87 ± 1.70	1.18 ± 2.31	0.529	0.003

Abbreviations: CART (Cocaine- and Amphetamine-Regulated Transcript), leptin (leptin gene expression), and eNOS (Endothelial Nitric Oxide Synthase). Gene expression levels were assessed prior to and six months following bariatric surgery. Values are presented as mean ± SD. Pearson correlation (r) values show the strength and direction of the link between pre- and post-surgical expression levels.

## Data Availability

Data are contained within the article.
